# Hyperspectral imaging with machine learning for non-destructive classification of *Astragalus membranaceus* var. *mongholicus*, *Astragalus membranaceus*, and similar seeds

**DOI:** 10.3389/fpls.2022.1031849

**Published:** 2022-11-29

**Authors:** Yanan Xu, Weifeng Wu, Yi Chen, Tingting Zhang, Keling Tu, Yun Hao, Hailu Cao, Xuehui Dong, Qun Sun

**Affiliations:** ^1^ College of Agronomy and Biotechnology, Department of Plant Genetics & Breeding and Seed Science/Chinese Medicinal Herbs Research Center, China Agricultural University/The Innovation Center (Beijing) of Crop Seeds whole-process Technology Research, Ministry of Agriculture and Rural Affairs/Beijing Key Laboratory of Crop Genetic Improvement, Beijing, China; ^2^ Key Laboratory of Ministry of Education for Genetics, Breeding and Multiple Utilization of Crops, College of Agriculture, Fujian Agriculture and Forestry University, Fuzhou, China; ^3^ Hengde Materia Medica (Beijing) Agricultural Technology Co., Ltd., Beijing, China

**Keywords:** Astragalus seeds, similar seeds, classification, machine vision, hyperspectral imaging

## Abstract

The roots of *Astragalus membranaceus* var. *mongholicus* (AMM) and A. membranaceus (AM) are widely used in traditional Chinese medicine. Although AMM has higher yields and accounts for a larger market share, its cultivation is fraught with challenges, including mixed germplasm resources and widespread adulteration of commercial seeds. Current methods for distinguishing Astragalus seeds from similar (SM) seeds are time-consuming, laborious, and destructive. To establish a non-destructive method, AMM, AM, and SM seeds were collected from various production areas. Machine vision and hyperspectral imaging (HSI) were used to collect morphological data and spectral data of each seed batch, which was used to establish discriminant models through various algorithms. Several preprocessing methods based on hyperspectral data were compared, including multiplicative scatter correction (MSC), standard normal variable (SNV), and first derivative (FD). Then selection methods for identifying informative features in the above data were compared, including successive projections algorithm (SPA), uninformative variable elimination (UVE), and competitive adaptive reweighted sampling (CARS). The results showed that support vector machine (SVM) modeling of machine vision data could distinguish Astragalus seeds from SM with >99% accuracy, but could not satisfactorily distinguish AMM seeds from AM. The FD-UVE-SVM model based on hyperspectral data reached 100.0% accuracy in the validation set. Another 90 seeds were tested, and the recognition accuracy was 100.0%, supporting the stability of the model. In summary, HSI data can be applied to discriminate among the seeds of AMM, AM, and SM non-destructively and with high accuracy, which can drive standardization in the Astragalus production industry.

## 1 Introduction

Astragalus commonly refers to *Astragalus membranaceus* var. *mongholicus* (AMM) or *A. membranaceus* (AM), a legume which is harvested for the medicinal properties of its roots, which are purported to confer anti-inflammatory, anti-oxidative, and anti-cancer effects (Bai et al., 2018; Chen et al., 2020; Zhang et al., 2021). Notably, Astragalus is among the 40 most commonly used traditional Chinese medicines, and its demand is continuously increasing.

However, the seed quality of traditional Chinese medicines is often markedly lower than that of other crops, with problems including seeds of mixed and unknown origins, contamination with debris and foreign matter, inconsistent maturity, low germination rates, and slow or irregular emergence. The adulteration of traditional Chinese medicines is common due to the high value and high demand for these products, which has aroused widespread concerns for public safety (Reid et al., 2006; Yang et al., 2021b). Because it is primarily propagated by seed, Astragalus production faces particular challenges, such as mixed germplasm resources, uneven seed quality, and substantial adulteration of commercial seeds. These cumulative factors adversely affect Astragalus cultivation, and present the challenge of distinguishing between Astragalus and similar (SM) seeds.

AMM and AM are both included in the 2020 edition of the Chinese Pharmacopoeia, and although *A. complanatus*, *Melilotus officinalis*, *A. sinicus*, and *Hedysarum polybotrys* are also considered authentic medicines, they are common SM seeds found as contaminants in AM or AMM seed lots. In addition to contamination with SM seeds, AM and AMM seeds are often mixed, which is problematic due to differences in growth habit, planting adaptability, cultivation and management techniques, chemical composition, and commercial value. AMM plants are morphologically shorter than AM, but the roots are typical taproot, with characteristically few root branches and high yield (since the root is the harvested portion). By contrast, AM plants are taller, with shorter roots that exhibit higher branch number, and lower yield (Wang and Liu, 1996; Zhang et al., 2009; Yang et al., 2020). Therefore, AMM is the most widely cultivated variety, and mixing AMM and AM seeds will lead to different heights during planting, which limits the effectiveness of AMM management practices and decreases yield. This ongoing problem in AMM cultivations points to a need for a system that can accurately sort AMM and AM seeds.

Conventional methods of distinguishing between AMM, AM, and SM seeds include observations with an electron microscope, physical and chemical methods, ultraviolet spectroscopy, and molecular labeling (Yan et al., 2001; Yan et al., 2005; Wang et al., 2005; Duan et al., 2012; Zheng et al., 2019). However, these methods generally produce qualitative results that depend heavily on experience, especially visual evaluation, and a non-destructive, accurate, and simple method for discriminating among AMM, AM and SM seeds is urgently needed by Astragalus producers and market regulators alike.

Machine vision technology combines computational analysis with image recognition and processing technologies (Savakar and Anami, 2009; Patel et al., 2012). Machine vision with image processing (typically RGB images) is currently widely used in agriculture, while research on seed classification is also developing (Cheng et al., 2010; Huang and Cheng, 2017; Tu et al., 2021). Although machine vision can identify morphological and textural variation well, it does not provide any non-visual trait data, such as internal composition, which has limited the application of this approach for distinguishing seeds from genetically and phenotypically similar varieties. This problem has been overcome through hyperspectral imaging (HSI), which simultaneously integrates spatial data with spectral information to highlight differences in chemical composition that affect light reflection or transmittance through the sample, providing additional analytical layers for each sample. In addition, HSI provides high spatial ​resolution, generating continuous and narrow-band spectral information for a given object (Guo et al., 2017; Paoletti et al., 2019). HSI techniques are increasingly tested for application in the identification of crops such as staple grains, fruits, and vegetables (Choudhary et al., 2009; Nansen et al., 2015; Sun et al., 2016; Wang et al., 2016; Wang et al., 2018; Xiao et al., 2020; Liu et al., 2022), supporting its feasibility for distinguishing different seed types.

It should be noted that analyzing the large datasets obtained by machine vision or HSI have presented a non-trivial challenge for data scientists and researchers. Machine learning methods have been developed that are currently the most efficient approaches for image processing and analysis. Common machine learning algorithms, including support vector machine (SVM), partial least squares discriminant analysis (PLS-DA), and multilayer perceptron (MLP), have been successfully applied to a range of classification tasks (Yang et al., 2015a; Sun et al., 2016; Sun et al., 2021; Nazari et al., 2021).

Machine vision and HSI approaches have been combined with machine learning algorithms to classify different crop seeds (Hong et al., 2016; Sun et al., 2017a; Zhao et al., 2018; Nie et al., 2019; Nazari et al., 2021; Ruslan et al., 2022; Tu et al., 2022). **Table 1** shows details of these classification tasks. For Astragalus, Xiao et al. (2020) used visible/short-wave near-infrared and near infrared hyperspectral imaging with a convolutional neural network to identify Radix Astragali from five geographical origins, showing an accuracy of >98%. Despite the success of machine vision and HSI technologies in different crop seeds, to our knowledge, no studies have yet reported the application of machine vision and HSI technologies in AMM, AM and SM seed identification.

**Table 1:** Application of machine vision and HSI for different crop seed classification tasks.

Method	Seed	Varieties	Classifier(s)	Result	Reference
Machine vision	Sorghum	10 sorghum cultivars	MLP, MLT	99%	Nazari et al., 2021
	Weedy rice	5 cultivated rice varieties and a weedy rice seed	LR	92.40%	Ruslan et al., 2022
	Rice	6 common cultivated rice seed varieties	KNN, SVM, RF	90.54%	Hong et al., 2016
HSI	Grape	3 grape seed varieties	SVM	94.30%	Zhao et al., 2018
	Maize	Jingke 968 and Non-Jingke 968	RF, SVM, MLP	~99%	Tu et al., 2022
	Hybrid okra seeds and hybrid loofah seeds	6 hybrid okra seed varieties and 6 hybrid loofah seed varieties	PLS-DA, SVM, DCNN	~95%	Nie et al., 2019
	Rice	Rice seeds from 4 different regions	SVM	91.67%	Sun et al., 2017a

In this study, machine vision and HSI techniques were applied to the identification of AMM, AM and SM seeds of different origins. The specific objectives of this work include: (1) to establish detection models for AMM, AM and SM seeds using machine vision or HSI image data combined with a machine learning algorithm; (2) to determine the optimal classification model based on the predictive accuracy of different models with the validation dataset; (3) to compare the detection accuracy of the strongest machine vision- or HSI-based models for sample seeds not included in the training data; (4) to compare machine vision and HSI methods to determine which imaging method is better suited for AMM, AM and SM seed identification.

## 2 Materials and methods

### 2.1 Materials

The seed samples used in this study were divided into “Astragalus seeds” and “SM seeds”. Astragalus seeds include AMM and AM seeds, while SM includes *Astragalus complanatus*, *Melilotus officinalis*, *A. sinicus*, and *Hedysarum polybotrys* seeds. After the seeds were collected, they were sealed and fumigated with aluminum phosphide for 4-5 days, placed in a ventilated place for 7-10 days, bagged, and stored in a ventilated place at room temperature. All samples were identified by field planting and their known origin. The source and quantity of seed samples collected in this study are shown in **Table 2**.

**Table 2:** Table of materials.

Category	Species	Identifier	Source	Genotypes	Number of seeds	
					Machine vision	Hyperspectral
Astragalus seeds	*A. membranaceus* var. *mongholicus* (AMM)	AMM-1	Tangshan, Hebei	Cultivated	234	50
		AMM-2	Datong, Shanxi	Cultivated	249	50
		AMM-3	Chifeng, Inner Mongolia	Cultivated	199	50
		AMM-4	Yulin, Shaanxi	Cultivated	215	50
		AMM-5	Guyuan, Ningxia	Cultivated	184	50
		AMM-6	Shangzhuang, Beijing	Cultivated	361	50
		AMM-7	Anguo, Hebei	Cultivated	85	50
		AMM-8	Market purchase	Cultivated	186	50
		AMM-9	Longnan, Gansu	Wild	234	50
	*A. membranaceus* (AM)	AM-1	Tangshan, Hebei	Cultivated	290	50
		AM-2	Zhangjiakou, Hebei	Cultivated	256	50
		AM-3	Hezheng, Gansu	Cultivated	237	50
		AM-4	Guyuan, Ningxia	Cultivated	197	50
		AM-5	Shangzhuang, Beijing	Cultivated	239	50
		AM-6	Longnan, Gansu	Wild	202	50
		AM-7	Zhangjiakou, Hebei	Wild	240	50
Similar (SM) seeds	*A. complanatus*	AC-1	Anguo, Hebei	Cultivated	427	50
		AC-2	Bozhou, Anhui	Cultivated	325	50
		AC-3	Suqian, Jiangsu	Cultivated	407	50
		AC-4	Laboratory samples	/	352	50
	*M. officinalis*	MO-1	Laboratory samples	/	132	50
	*A. sinicus*	AS-1	Laboratory samples	/	214	50
	*H. polybotrys*	HP-1	Laboratory samples	/	145	50

### 2.2 RGB image acquisition and feature extraction

Several AMM, AM, and SM seeds were randomly selected and scanned with a ScanMaker i360/i460 scanner (Shanghai, China) at a resolution of 600dpi. The images were saved in TIFF lossless format. Hundreds of seeds were scanned each time, though there was no contact between seeds.

The Phenoseed automatic extraction system, which was jointly developed by the Seed Science and Technology Research Center of China Agricultural University (Beijing, China) and Nanjing Zhinong Yunxin Big Data Technology Co., Ltd. (Nanjing, China), was used to extract phenotypic features of the seeds. Shape features included length (mm), width (mm), L/W ratio, area (mm2), perimeter (mm), and roundness (mm). Color features included R (red in the primary color light spectrum), G (green in the primary color light spectrum), B (blue in the primary color light spectrum), L (luminosity), a (range from red to green), b (range from blue to yellow), hue, saturation, value, gray, and standard deviation. Texture features included the average value and standard deviation of contrast, dissimilarity, homogeneity, energy, correlation, ASM, and entropy under gray, R, G, and B. There were a total of 54 phenotypic features.

### 2.3 Hyperspectral reflectance data extraction

#### 2.3.1 HSI system and analysis software

Hyperspectral reflectance images of seeds were collected using a prototype visible/near-infrared (VIS/NIR) HSI system installed at the Beijing Key Laboratory of Crop Genetic Improvement, China Agricultural University. The spectral range of the system was 311- 1090 nm, the bandwidth was 0.78 nm, and the image resolution was 1004 × 1002 pixels. The software for collecting spectral information was Spectral Image-VNIR (Isuzu Optics Corp., Taiwan, China). Other detailed descriptions of the VIS/NIR HSI system are listed in a paper published by Zhang et al. (2020).

#### 2.3.2 Hyperspectral image acquisition and data extraction

Before collecting hyperspectral images, we calibrated the black-and-white board to produce the corrected image and then set the moving speed of the electric control platform to 1.7 mm/s and the exposure time of the camera to 6 ms. When collecting spectral information from the sample, we placed each seed on the electric control displacement table. The camera scanned the whole platform as it moved. We then collected 50 seeds from each batch, for a total of 1150 seeds. The hyperspectral image acquisition process was completed in the dark box.

Before extracting spectral information, an HSI analyzer (Isuzu Optics Corp., Taiwan, China) was used to correct the spectral image, as shown in equation (1), where *I* is the corrected hyperspectral image. I_0_ is the original hyperspectral image; B is the blackboard file image (reflectivity close to 0%), and W is the whiteboard file image (reflectivity close to 100%).


(1) $$I=\frac{{\text{I}}_{0}-\text{B}}{\text{W}-\text{B}}\;\;\;\;\;\;\;\;\;\;\;\;\;\;\;\;\;\;\;\;\;\;\;\;$$

After the black-and-white plate was corrected, the single seed was separated from the background of the hyperspectral image. The background and seeds were separated by setting a threshold and obtaining the region of interest (ROI) by morphological filtering and mask processing. Due to the large noise interference in the head and tail bands, only the reflection spectra of 765 bands within 400-1000 nm of each seed were extracted for subsequent modeling and analysis. Among them, the VIS included 490 reflectance data points in the range of 400-780 nm, and the NIR included 275 reflectance data points in the range of 780-1000 nm.

### 2.4 Spectra preprocessing

Spectral information is inevitably affected by various factors, and spectral preprocessing is used to improve the usefulness of spectral data (Esquerre et al., 2012). This study compared three spectral preprocessing methods, including multiplicative scatter correction (MSC), standard normal variable (SNV), and first derivative (FD). From the latter, MSC and SNV were used to consider the addition/multiplication effect and scattering effect in spectral data (Silalahi et al., 2018; Wu et al., 2019). FD helps delete baseline offset (Qu and Liu, 2017). These preprocessing methods eliminate the external interference generated during the acquisition of hyperspectral images. In this study, a suitable preprocessing method was selected based on the preprocessing effect of various preprocessing methods on the raw spectral data.

### 2.5 Selection of effective wavelengths

The presence of high dimensional data and a large amount of redundant information in hyperspectral images can affect the modelling speed. Therefore, it is important to use the variable selection method during the analysis and processing of hyperspectral data. In this study, successive projection algorithms (SPA), uninformative variable elimination (UVE), and competitive adaptive reweighted sampling (CARS) were used to select EWs.

SPA can extract low collinearity and low redundancy variables to avoid the influence of information overlap and collinearity. When the SPA method is used to optimize the wave band, multiple linear regression models can be established one by one for different wave segment subsets, and the root means square error (RMSE) value can be calculated, in which the number of variables corresponding to the lowest RMSE is the optimal EWs (Galvão et al., 2008; Zhang et al., 2018b). UVE can remove the wavelength variables that contribute less to the modeling and select the characteristic wavelength variables. The removed wavelength variables are called non-information variables. UVE and CARS establishment are based on the PLS algorithm. To select non-informative variables, the UVE algorithm adds a group of white noise variables with the same number of original variables to the PLS model and obtains the regression coefficient corresponding to each variable based on the cross-leave method of the PLS model. The stable value of each variable coefficient is divided by the standard deviation, their quotient is compared with the stable value obtained from the random variable matrix, and those wavelength variables that are invalid for modeling are deleted as random variables. (Put et al., 2006; Wang et al., 2020) In CARS algorithm, adaptive weighted sampling (ARS) is used to retain the points with large absolute values of regression coefficients in the PLS model as a new subset each time, and remove the points with small weights. Then, the PLS model is established based on the new subset. After multiple calculations, the subset with the smallest cross-validation root mean square error (RMSECV) of the PLS model is selected as the characteristic wavelength. (Zhang et al., 2018a; Zhou et al., 2020a)

### 2.6 Data−driven modeling

Support vector machine (SVM), partial least squares discriminant analysis (PLS-DA), and multilayer perceptron (MLP) are widely used classification methods that have been well validated for seed detection (Kujawa et al., 2014; Nazari et al., 2021; Yang et al., 2021a). In this study, three classification models of AMM, AM, and SM seeds were established using SVM, PLS-DA, and MLP, respectively. SVM is widely used to solve linearly differentiable and linearly indistinguishable classification problems, and the radial basis kernel function (RBF) kernel is the most common and effective method for classification problems. Optimization of the hyperparameters is necessary before the real model can be trained. In current practical applications, hyperparameters are usually determined empirically or by grid search. (Liu et al., 2022) PLS-DA is a typical classification method, which is considered as a supervision method to distinguish samples to the maximum extent. (Nie et al., 2019; Zhang et al., 2022b) MLP is a feedforward neural network. It maps a set of input vectors to a set of output vectors. The inputs and outputs can be connected by multilayer weighting, with strong self-learning, adaptive, associative memory and parallel processing of things and environments. (Xu et al., 2021)

To avoid the effect of default parameters on the prediction accuracy of the classification model, the internal parameters of the classification model must be separately adjusted. In the SVM algorithm, the RBF kernel was selected, and it carried out the 5-fold internal cross-validation and grid search method to calculate optimal penalty coefficient c and the kernel parameter g. The searching range was both set to -10 to 10 with the step of 0.2 (a total of 101*101 combinations were used to search the best parameters). In the PLS-DA model, the number of latent variables (LVs) changes, and the model correctly identifies the highest percentage of seeds. The MLP network with two hidden layers was selected, and the hidden layer adopted the hyperbolic tangent activation function of SPSS. The output layer adopted the Softmax activation function.

### 2.7 Analyzing

**Figure 1:**
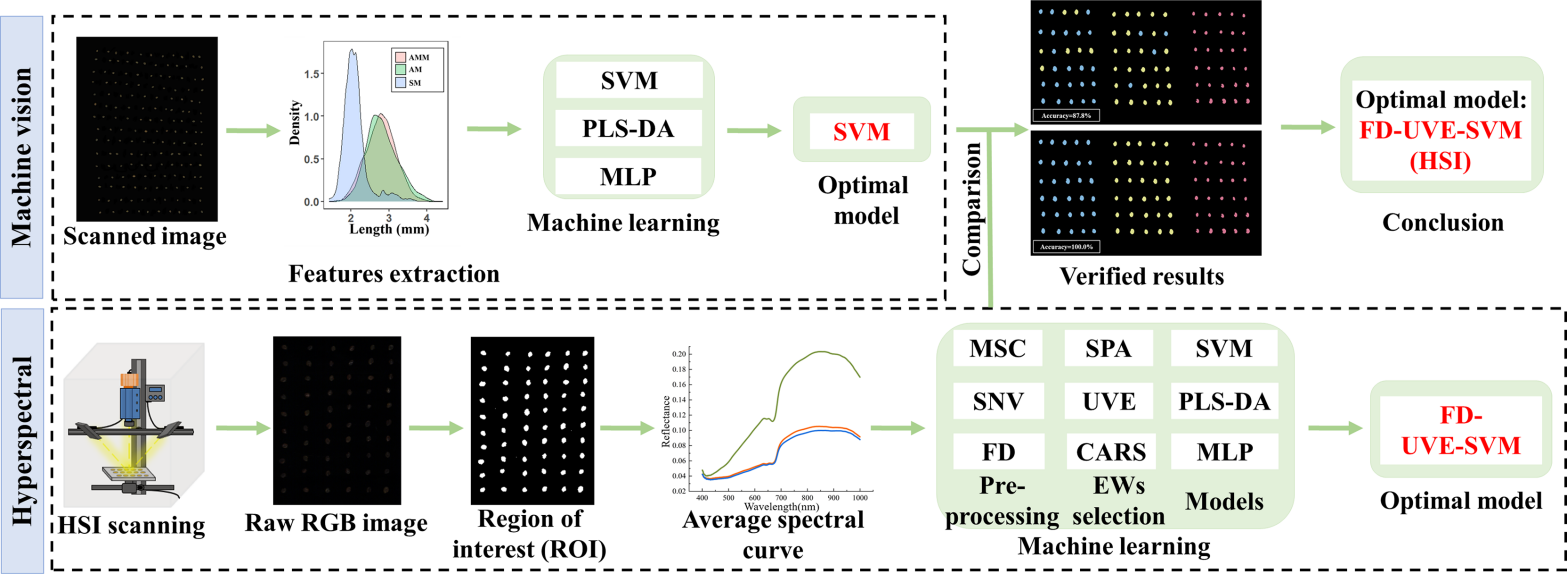
Technical routes.

MSC, SNV, FD spectral preprocessing, SPA, UVE, CARS feature band extraction, and SVM and PLS-DA model driving processes were implemented in Matlab R2020b. The MLP modeling process was using IBM SPSS statistics 26. For each model, the ratio of three model training sets and validation sets was 7:3. The specific quantity of seeds in each batch is shown in **Table 2**. Origin 2022 and R 4.1.2 were used to visualize data. The experimental flow of this study is shown in **Figure 1**.

## 3 Results

### 3.1 Identification of AMM, AM, and SM seeds based on machine vision

**Figure 2:**
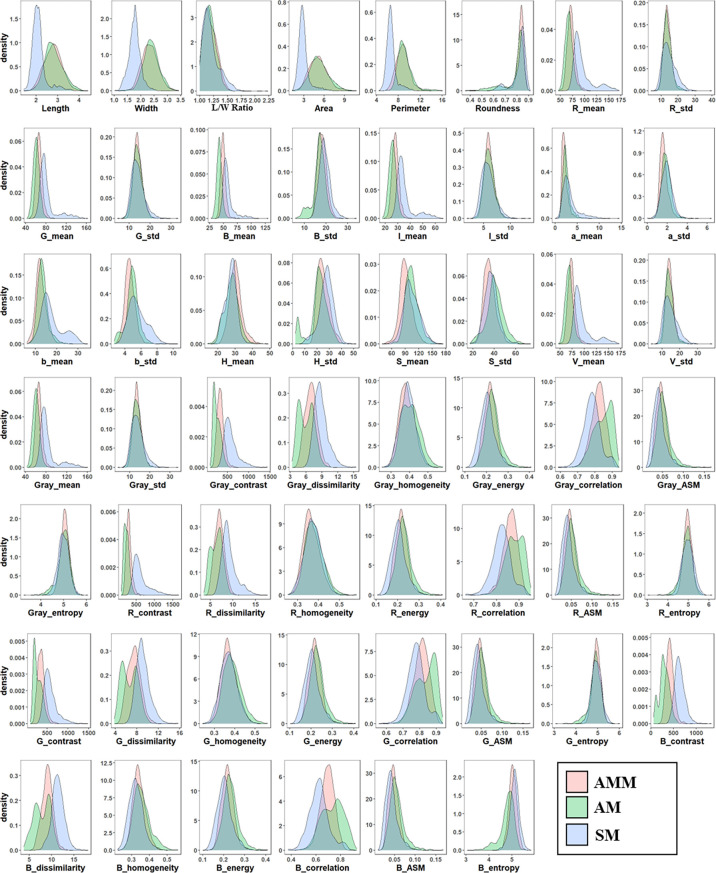
Probability density distribution of phenotypic features of AMM, AM, and SM seeds.

In order to develop a machine learning algorithm capable of distinguishing AMM, AM, and other SM seeds, we first tested untrained machine vision using a mixed set of 5610 seeds. The machine vision technology based on Phenoseed automatic extraction system extracted a set of 54 potentially informative morphological features for discriminating among seed types, including shape, color, and texture. A probability density map was then generated to examine the distributions of these phenotypic features across the 5610 combined AMM, AM and SM seeds (**Figure 2**), which showed high overlap in their features related to color and texture, especially between AMM and AM. These results indicated that these features might not be sufficiently different between seed types to form a basis for distinguishing between them. It is worth noting that the size of SM was generally smaller than that of AMM or AM seeds, although the size distributions showed substantial overlap (**Figure 2**). Thus, SM resembled small AMM and AM seeds, which is germane to distinguishing SM seeds mixed into Astragalus seed lots in the market.

**Figure 3:**
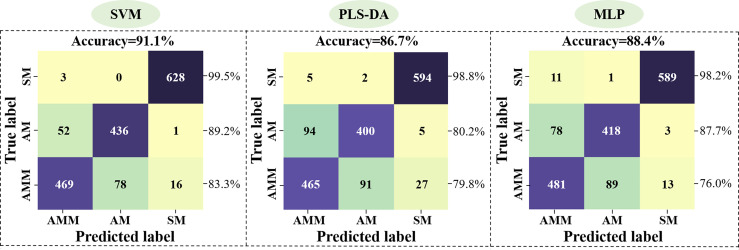
Performance of the SVM, PLS-DA, and MLP model validation sets based on machine vision data.

In order to establish a model for classifying AMM, AM, and SM seeds, the 54 morphological features detected by machine vision were used as inputs for SVM, PLS-DA, and MLP networks, with 3927 seeds in the training set and 1683 seeds in the validation set. The accuracy in distinguishing between seeds was then calculated for each model (**Figure 3**), with 48 latent variables (LVs) selected for the PLS-DA model. Classification accuracy of SM seeds using these features reached >98.2%, but the classification effect of AMM and AM seeds were not ideal. In particular, SVM showed 83.3% accuracy for identifying AMM seeds, 89.2% accuracy for detecting AM seeds, and 99.5% accuracy for SM seeds, resulting in 91.1% average accuracy for this model.

### 3.2 Identification of AMM, AM, and SM seeds using HSI

Since machine vision with different machine learning algorithms could not effectively distinguish AMM seeds from AM seeds, we next explored means of improving accuracy by VIS/NIR hyperspectral imaging of reflectance spectra between 400 nm and 1000 nm bands for each seed type.

#### 3.2.1 Spectral characteristics

**Figure 4:**
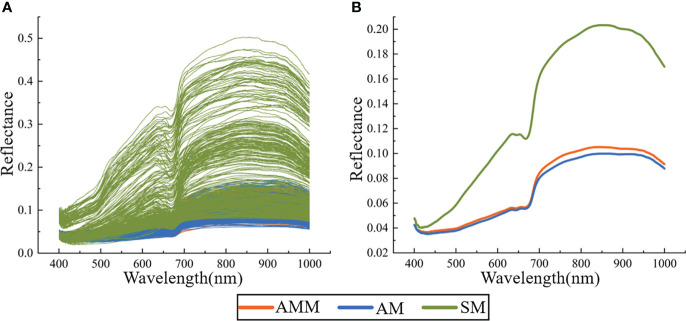
Reflection spectrum **(A)** and average spectrum **(B)** of AMM, AM, and SM seeds.

To identify wavelengths that were distinct among seed types, the reflectance spectra were obtained for 1150 seeds (**Figure 4A**) and the average spectrum of AMM, AM, and SM seeds were calculated (**Figure 4B**), which revealed that the average spectra of AMM and AM seeds was significantly lower than that of SM seeds. By contrast, AMM and AM seeds displayed similar spectra, which agreed well with previous studies examining the variety and viability of seeds from other crops (Yang et al., 2017; Wakholi et al., 2018; Yang et al., 2021a). However, the reflectance of AMM was generally higher than that of AM seeds, with greater differences in the NIR region (780-1000 nm) than in the VIS region (400-780 nm). The VIS region may be related to β-carotene and anthocyanin in the seeds (Sun et al., 2021), and the difference in the NIR region may be related to protein, starch and other organic matter in the seeds (Cen and He, 2007; Awanthi et al., 2019). These results indicated that HSI could capture differences in texture, pigment and other physical and chemical properties between AMM and AM seeds. And the differences between AMM and AM seeds in terms of organic matter such as protein and starch are greater than the differences in pigment content, which explains the inability of models based on VIS data (machine vision) to correctly distinguish between AMM and AM. Further analysis showed that in the range of 660-750 nm, the spectral curve showed an obvious upward trend, and the average spectral curve of the two seeds gradually separated. The reason is that this wavelength corresponds to the vibration of N-H chemical bond of amino acid in seeds, which can be used to verify the difference of amino acid content in AMM and AM seeds. (Yang et al., 2021a; Wang et al., 2022) In addition, there are four absorption peaks in the average reflectance spectra of AMM and AM seeds (valleys at 415 nm, 640, 680 and 885 nm). The carotenoid (Yang et al., 2021a) and proanthocyanidin content of the seed coat (Wang et al., 2022) can be determined at about 415 nm; The bands at about 640 nm and 680 nm may be associated with the absorption of chlorophyll b and chlorophyll a (Zhang et al., 2016). The band at about 885 nm may be associated with C-O, N-H, C-H and O-H bonding vibrations in proteins, carbohydrates and fats (Caporaso et al., 2018). However, the spectral curves were not unique enough to distinguish AMM, AM, and SM seeds. More specifically, while a large proportion of SM overlapped with Astragalus seeds in the original spectral curve, the reflectance spectra of AMM and AM showed extremely high overlap, making them indistinguishable by curve shape. To identify specific spectral features or effective wavelengths that could be used for classification of AMM, AM, or SM seeds, it was first necessary to generate discriminant analytical models to test different methods of preprocessing to maximize the accuracy of discriminating among AMM, AM and SM seeds.

#### 3.2.2 Spectral preprocessing and effect analysis

**Figure 5:**
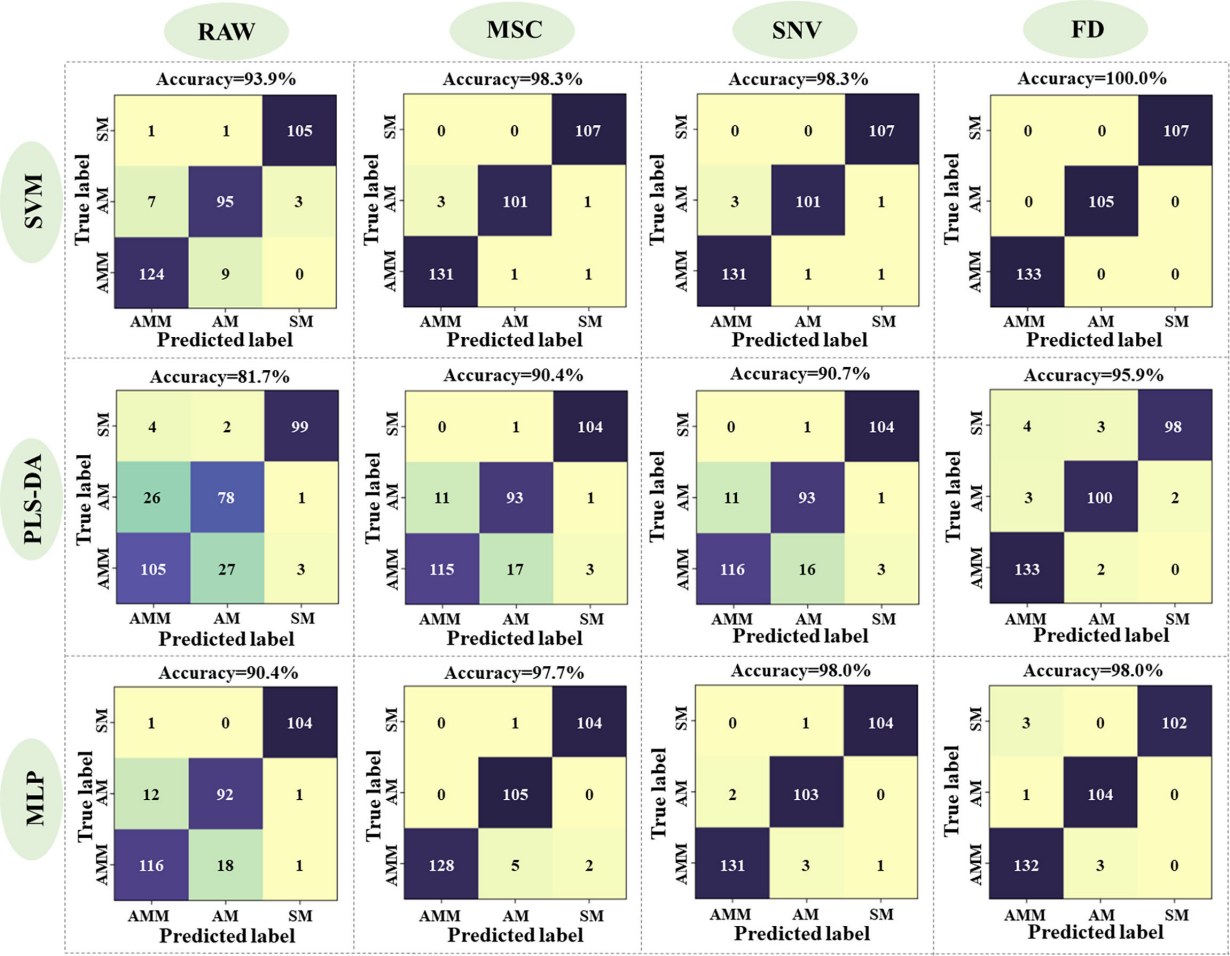
Accuracy of SVM, PLS-DA, and MLP models with spectral data that was preprocessed or not.

In order to establish the most effective discriminant model, we first tested three methods for preprocessing the seed reflectance spectra, including MSC, SNV and FD, as well as raw data. The SVM, PLS-DA, and MLP methods were each used to generate models with the processed and raw data, using a training set of 805 seeds and a validation set of 345 seeds (i.e., a 7:3 ratio) (see section 2.6 for parameter details). The PLS-DA model selected 10, 10, 10, and 9 LVs from raw spectra or spectra preprocessed by MSC, SNV, FD, respectively. Validation of each classifier model with each respective preprocessing method showed that accuracy ranged from 81.7%, in the PLS-DA model with raw data, to 100.0% in the SVM model with the FD-processed spectra (**Figure 5**). Among the three pre-processing methods, the model built after FD pre-processing was the optimal, while MSC and SNV showed suboptimal accuracy compared to FD, but still better than RAW. Among the three classifiers, the performance of the model showed SVM > MLP > PLS-DA, regardless of the pre-processing based. While accuracy differed to a limited extent between each combination of classifier and data processing method, it warrants mention that all preprocessing methods increased the accuracy over that of raw data input for all classifiers by denoising the original reflectance spectra. Since the FD-SVM denoising/classifier combination provided the highest accuracy in discriminating among AMM, AM, and SM seeds, this method was used in subsequent analyses of effective wavelengths HSI reflectance data.

#### 3.2.3 Selection of effective wavelengths

Based on the above results showing highly accurate classification of AMM, AM, and SM seeds obtained by full spectra models, we next sought to increase computational efficiency and reduce processing time by identifying effective wavelengths in FD-processed data that were informative for classifying seed types.

**Figure 6:**
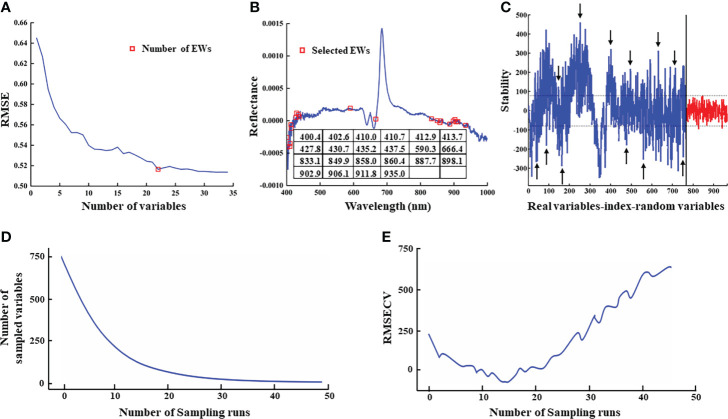
Results of EWs selection. **(A)** RMSE for the number of EWs by SPA; **(B)** The position of EWs identified by SPA in FD-processed spectrum; **(C)** EWs selected by UVE. Peaks in the blue curve represent stability values for the 765 wavelengths; the red curve shows the distribution of stability values of random noise variables generated by UVE which were used to determine the thresholds. The two horizontal dashed lines indicate the upper and lower thresholds of band selection. Bands between the thresholds were eliminated as invalid information, and the remaining variables were selected. The arrowheads show a few representative wavelengths; **(D)**Variation of wavelength variables number by CARS; **(E)** Variation of RMSECV by CARS.

Since the algorithmic principles underlying wavelength selection can impact modeling results (Zhang et al., 2020), we tested three approaches to EW extraction, SPA, UVE, and CARS. In SPA extraction, the root means square error (RMSE) values decreased with increasing number of variables (i.e., EWs). The lowest RMSE value coincided with 22 variables, above which RMSE remained stable (**Figure 6A**). Therefore, an RMSE threshold of 0.5164 with 22 effective wavelengths in the denoised reflectance spectrum was selected for subsequent tests (**Figure 6B**). By contrast, UVE analysis identified 391 potentially informative EW features (**Figure 6C**) for classifier analysis. In CARS extraction, the number of EWs decreased rapidly with the exponential decay function, but decreased at a slower rate as the number of samples increases (**Figure 6D**). The cross-validation root mean square error (RMSECV) tended to decrease and then increase as the number of samples increased, with the smallest RMSECV value when the number of samples reached 14 (**Figure 6E**) (i.e., the subset of EW selected for this sampling was the key variable for predicting AMM, AM and SM seeds). Therefore, 140 potentially informative EW features (**Figure 6D**) were identified for classifier analysis after CARS.

The EWs selected by SPA were further examined to better understand their relevance as a theoretical basis for distinguishing AMM, AM and SM seeds. Several EWs were located around specific regions, which suggested that seed reflectance was related to the presence of specific chemicals. Some specific wavelengths in the visible light spectrum are reportedly related to plant pigments, such as absorption peaks for carotenoids at 412.9 and 413.7 nm, chlorophyll a at 427.8, 430.7, and 666.4nm, and anthocyanin at around 435.2 and 437.5 nm (Li et al., 2013; Nansen et al., 2015; Yang et al., 2015b; Zhang et al., 2016). Among the EWs screened by SPA, the selection of bands near pigment absorption peaks indicated that accurate classification of AMM, AM, and SM seeds may rely on seed color. By contrast, several EWs in the NIR were attributable to various chemical bonds. For example, EWs located near 833.1 nm were related to the vibrations of C-O, N-H, C-H, and O-H bonds in proteins, carbohydrates, and fats, respectively (Cen and He, 2007; Sun et al., 2017b; Caporaso et al., 2018). Tannin absorption peaks were also detected near 887.7 and 898.1 nm, and cellulose absorption peaks were present near 935.0 nm (Wang et al., 2022). In addition, peaks between 860-970 nm reflected the vibration of N-H bonds in proteins and amino acids, and suggested that these seeds differed in protein or amino acid contents (Zhou et al., 2020b). Since tannin, cellulose, and protein contents in the seed coat affect the thickness and hardness of the seed coat, these results indicated that AM, AMM, and SM seeds differed in their seedcoat hardness.

#### 3.2.4 SVM modeling based on different EW selection methods

**Figure 7:**
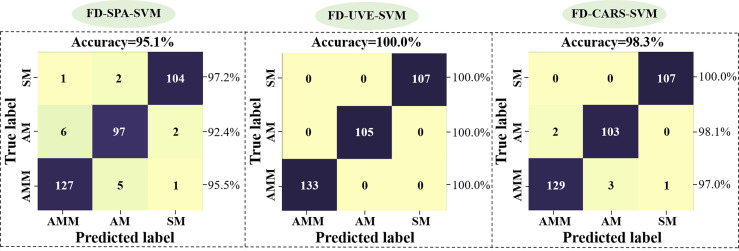
Validation of SVM model accuracy based on EW data selected by SPA (left), UVE (middle) or CARS (right) methods.

In light of our above findings, we next tested the accuracy of the SVM model in discriminating between AM, AMM, and SM seeds using FD-denoised EWs selected by SPA or UVE or CARS as inputs. Validation of model accuracy is shown in **Figure 7**. The changes in model performance indicate that UVE screening of EWs results in higher accuracy, with CARS showing the sub-optimal accuracy and SPA the worst performance. It potentially due to an insufficiently large sample set of EWs selected by SPA, leading to the exclusion of informative wavelengths related to seed classification. Our results thus showed that UVE method is more suitable than the SPA and CARS algorithm for building SVM models to classify AMM, AM, and SM seeds and that the FD-UVE-SVM combination provides an optimal model for this task, providing 100.0% accuracy in the validation set.

### 3.3 Visual comparison between machine vision and HSI prediction results

**Figure 8:**
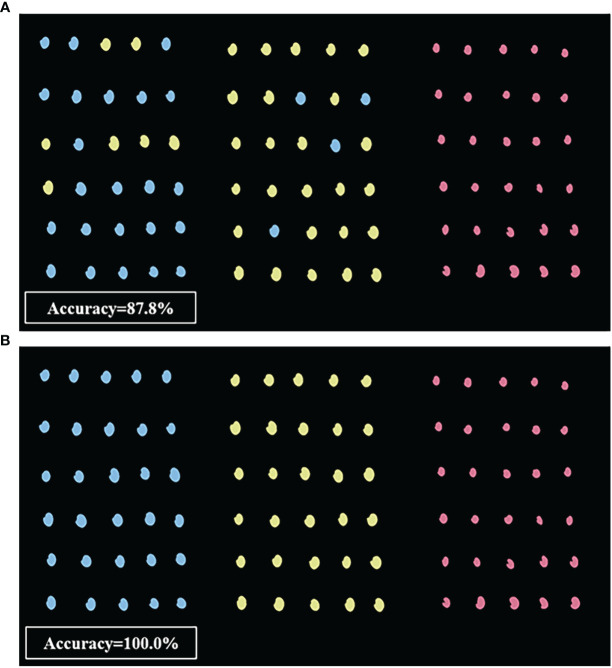
Visualization results based on **(A)** machine vision optimal model and **(B)** HSI optimal model (from left to right: AMM, AM, and SM seeds).

Based on the above results showing that SVM classification provided the highest accuracy with machine vision imaging data, while the FD-UVE-SVM model showed the highest accuracy with HSI-based data, we next visually examined a set of 90 seeds, including 30 AMM, 30 AM, and 30 SM, to manually verify the classifications made by these two models (**Figure 8**). The SVM model based on machine vision image data (**Figure 8A**) could correctly predict the 30 SM seeds, while 7 of the 30 AMM seeds were incorrectly predicted as AM seeds, and 4 of the 30 AM seeds were incorrectly predicted as AMM seeds (i.e., an overall accuracy of 87.8%), indicating sub-optimal discriminatory power between AM and AMM seeds. By contrast, FD-UVE-SVM modelling of HSI data correctly predicted the types of all 90 seeds (i.e., 100.0% accuracy), indicating that hyperspectral data contained sufficient information to accurately discriminate between AMM, AM, and SM seeds, supporting its application in routine analysis required for seed sorting and quality control in production settings.

## 4 Discussion

In this study, HSI data outperformed machine vision image data in SVM-based models for distinguishing AMM, AM and SM seeds, which aligns well with studies comparing these approaches for classifying kernels of rice (Fabiyi et al., 2020) and maize (Tu et al., 2022), as well as comparing chlorophyll content in sorghum leaves (Zhang et al., 2022a). It is likely that machine vision data resulted in lower accuracy in classifying AMM and AM seeds because the features extracted by machine vision were purely morphological phenotypes. Since AMM and AM belong to the same species, they share highly similar seed morphology. Thus, other phenotypic data, such seed traits related to the internal accumulation or deposition of specific metabolites affecting light reflection or diffraction captured by HSI data can provide more informative features for discriminating among subspecies. Although both methods are non-destructive and high-throughput tests that enable screening of intact seeds, each technology is accompanied by advantages and disadvantages. The main advantages of machine vision over HSI are the relatively low instrument cost and faster image acquisition, which are linked to its main disadvantage of capturing strictly morphological information that cannot account for many internal, physiological seed traits. By contrast, HSI data includes hundreds or thousands of spectral bands, and therefore contains more information allowing more robust discrimination among samples. However, it should be pointed out that the high dimension of spectral data limits the calculation speed and processing time to some extent. Moreover, HSI equipment is relatively expensive and the operating cost is high.

In HSI data analysis, FD pre-processing achieved superior results to MSC and SNV, which aligns well with studies for classifying kernels of sugar beet (Yang et al., 2018) and the detection of germination rates in sorghum-sudan grass seed (Hui et al., 2022). It is likely that due to FD eliminates baseline drift in AMM, AM and SM seed spectral data and improves the spectral band characteristics and spectral resolution, thus FD pre-processing achieves the highest accuracy (Qu and Liu, 2017; Li et al., 2021). However, it is worth noting that spectral derivatives can also increase the noise level and reduce the spectral signal-to-noise ratio, which is detrimental to modeling. The higher the derivative order, the more serious the degradation of the signal-to-noise ratio. Therefore, in spectral analysis, only FD or second derivative (SD) is generally used for spectra.

Selecting a subset of EW features by SPA or UVE or CARS to construct the model can dramatically reduce processing time (Gao et al., 2013). SPA-based screening of denoised EWs captures a portion of bands in the NIR wavelength range (>780nm), whereas machine vision technology collects wavelengths largely in the VIS range. These additional NIR EWs may explain why HSI technology can provide higher accuracy than machine vision in distinguishing AMM, AM, and SM seeds.

Although HSI technology has enormous potential for discrimination among highly (visually) similar seeds, sample sizes in this study, including nine batches of AMM, seven batches of AM, and seven batches of SM, were not adequately large for robust statistical verification. Follow-up research will necessarily include larger sample sets improve the generalizability and accuracy of the classification model. In addition, in order to maintain model validity when testing seeds from different origins and growing seasons, the model can be updated using a method established for updating a maize seed detection model (Guo et al., 2017; Tu et al., 2022).

## 5 Conclusion

We compared machine vision and HSI image data to classify AMM, AM, and SM seeds, which led to the following specific conclusions:

1) SVM-based models of machine vision image data to distinguish AMM, AM, and SM seeds indicated that AM/AMM seeds could be distinguished from SM seeds at >99.0%, but could not well-distinguish between AMM and AM seeds.
2) The application of a FD-UVE-SVM model to HSI data resulted in 100.0% accuracy, thus validating the SVM classification model as the best suited for distinguishing SM, AM, and AMM seeds.
3) Verification of model accuracy based on machine vision and HSI data from a 90-seed verification set indicated that predictive accuracy was 100.0% with HSI data, demonstrating the efficiency, reliability, and simplicity of this model, and importantly, revealing that HSI is more suitable for discriminating among AMM, AM, and SM seeds.
In general, this study used machine vision and HSI technology to classify AMM, AM, and SM seeds. FD-UVE-SVM modeling of HSI data can be used to accurately distinguish AMM, AM, and SM. This strategy can be adapted for routine analyses in production facilities. These advances can in turn increase the economic benefits of Astragalus seeds.

## Data availability statement

The original contributions presented in the study are included in the article/**Supplementary Material**. Further inquiries can be directed to the corresponding authors.

## Author contributions

QS, XD, and YX designed the experiments. YX collected the RGB, HSI images, analyzed the data, and wrote the manuscript. WW, YC, TZ, KT, YH, and HC provided comments and suggestions for the manuscript and checked the writing. All authors contributed to the article and approved the submitted version.

## Funding

This work was supported by the Datong Municipal Government [grant number 201904710111639]; and the National Administration of Traditional Chinese Medicine State Administration of Traditional Chinese Medicine [grant number 202004610111024].

## Conflict of interest

Author HC was employed by Hengde Materia Medica Beijing Agricultural Technology Co., Ltd.

The remaining authors declare that the research was conducted in the absence of any commercial or financial relationships that could be construed as a potential conflict of interest.

## Publisher’s note

All claims expressed in this article are solely those of the authors and do not necessarily represent those of their affiliated organizations, or those of the publisher, the editors and the reviewers. Any product that may be evaluated in this article, or claim that may be made by its manufacturer, is not guaranteed or endorsed by the publisher.

## Abbreviations

AMM, *Astragalus membranaceus* var. *mongholicus*; AM, *A. membranaceus*; SM, Similar; SVM, Support vector machine; HSI, Hyperspectral imaging; MSC, Multiplicative scatter correction; SNV, Standard normal variable; FD, First derivative; SPA, Successive projections algorithm; UVE, Uninformative variable elimination; CARS, Competitive adaptive reweighted sampling; PLS-DA, Partial least squares discriminant analysis; MLP, Multilayer perceptron; VIS/NIR, Visible/near-infrared; ROI, region of interest; EWs, Effective Wavelengths; RMSE, The root means square error; LVs, latent variables.
